# *Leishmania *IFT140 mutants show normal viability but lack external flagella: a tool for the study of flagellar function through the infectious cycle

**DOI:** 10.1186/2046-2530-4-S1-P49

**Published:** 2015-07-13

**Authors:** T Fowlkes-Comninellis, S Beverley

**Affiliations:** 1 Molecular Microbiology, Washington University-St. Louis, St. Louis, MO, USA

## 

A striking feature of the parasitic protozoan *Leishmania *is the dramatic remodeling of the flagellum during the infectious cycle; in the sand fly midgut the promastigote form has a long, motile flagellum, while within the phagolysosome of the mammalian macrophage the flagellum barely protrudes past the surface of the amastigote form. The flagellum functions in promastigote motility and likely in cell morphogenesis, division, and maintenance of flagellar pocket structure for both forms. The flagellar pocket is the sole site of endo- and exocytosis in trypanosomatids and a sensory role for the amastigote flagellum has been proposed but a function is not established. Intraflagellar transport (IFT) is required for flagellar assembly and viability in the long, motile flagella of trypanosomes but unknown in *Leishmania*. We asked whether IFT is essential for viability in 'long' promastigote and 'short' amastigote flagella in *L. donovani *strain Bob by targeting a core retrograde pathway gene *IFT140*. Using a plasmid segregation knockout approach [1], viable knockout promastigotes were readily obtained. *Δift140 *lack external flagella by light and scanning electron microscopy, and have defective axonemes by transmission electron microscopy, which was reversed by complementation. This is the first example of a *Leishmania *mutant lacking flagella while retaining normal viability and growth. Thus, *Δift140 *allows for further studies of promastigote flagellar function. In addition, *Ld*Bob can generate amastigotes *in vitro *and infect mammalian hosts allowing us to probe the effect of *Δift140 *on the short flagella of amastigotes and parasite virulence.
